# Experimental Study on One-Way BFRP Bar-Reinforced UHPC Slabs under Concentrated Load

**DOI:** 10.3390/ma13143077

**Published:** 2020-07-10

**Authors:** Xiaoqing Xu, Zhujian Hou

**Affiliations:** 1 School of Civil Engineering, Chongqing University, Chongqing 400045, China; 2 Key Laboratory of New Technology for Construction of Cities in Mountain Area (Chongqing University), Ministry of Education, Chongqing 400045, China; 3 School of Civil Engineering, Chongqing Jiaotong University, Chongqing 400045, China

**Keywords:** ultra-high performance concrete, fiber-reinforced polymer, one-way slab, punching shear, fiber bridging effect, ductility

## Abstract

The application of fiber-reinforced polymer (FRP) bars and ultra-high performance concrete (UHPC) in the field of civil engineering is promising. An innovative FRP bar-reinforced UHPC short-ribbed bridge deck slab, with low self-weight and high structural performance, was proposed in this study. The behavior of one-way basalt FRP (BFRP) bar-reinforced UHPC slabs under concentrated load was experimentally investigated, and compared with that of a steel bar-reinforced UHPC slab. The ultimate capacity of the one-way BFRP bar-reinforced UHPC slab was 0.59 times that of the steel bar-reinforced UHPC slab, while its ductility was better. Increasing the reinforcement ratio and loading area was beneficial to the ductility of one-way BFRP bar-reinforced UHPC slabs. Moreover, the model proposed by EI-Gamal et al. was found to be suitable for evaluating the punching shear capacities of one-way BFRP bar-reinforced UHPC slabs. However, the model failed to consider the unique strain-hardening characteristics of UHPC, which led to conservative prediction.

## 1. Introduction

For steel bar-reinforced concrete structures exposed to harsh environments, the corrosion of steel bars can lead to serious deterioration in structural performance and necessitate expensive rehabilitation. Fiber-reinforced polymer (FRP) bars possess numerous outstanding properties, such as their light-weight, high-strength, noncorrosive nature, and their magnetic transparency [1]. They have been used to replace steel bars in concrete members. The advantages of FRP bars for corrosion resistance have been proven via their applications in bridge, marine and underwater structures in aggressive environments [2,3,4], and several design guides are available [5,6,7]. In addition, the application of FRP bars is sometimes indispensable in structures where electromagnetic interference is problematic, such as in reinforcing the concrete foundations of magnetism observation stations, and in curtain walls to prevent electromagnetic disturbance or damage [4].

FRP bars have a lower elastic modulus than steel bars. As a result, FRP bar-reinforced normal concrete (NC) members generally exhibit greater deflection and crack width, and encounter difficulties in satisfying the requirements of the serviceability limit state. To solve this problem, a high reinforcement ratio is generally adopted, which, however, increases the cost and decreases the efficiency of the high-strength characteristics of FRP bars at the ultimate limit state. Using FRP bars together with steel bars has been verified to be an alternative solution to improving the structural performance of FRP bar-reinforced NC members [8]. However, the corrosion of steel bars in these FRP bar-reinforced NC members would be a major durability concern again. In recent years, using ultra-high performance concrete (UHPC) with FRP bars has been suggested by researchers [9,10,11] in order to satisfy the requirements for FRP bar-reinforced concrete members at the serviceability limit state. UHPC is an innovative cement-based engineering material, which was developed based on the ideas of eliminating coarse aggregates and optimizing particle packing density [12,13,14]. Compared with NC, UHPC exhibits unique strain-hardening characteristics, ultra-high compressive strength, superior toughness, and durability [15]. Ferrier et al. [9] firstly proposed and tested the FRP bar-reinforced UHPC beam. It was found that FRP bar-reinforced UHPC beams exhibited high bending stiffness, and the short steel fibers prevented the degeneration of microcracks into macrocracks in UHPC. Yoo et al. [10,11] noted that GFRP bar-reinforced UHPC beams exhibited very high post-cracking stiffness, and satisfied the crack width requirement at the serviceability limit state as a result of the strain-hardening characteristics of UHPC. It is believed that FRP bar-reinforced UHPC structures are possible, combining the unique advantages of FRP bars and UHPC.

## 2. Background

### 2.1. The Proposed FRP Bar-Reinforced UHPC Short-Ribbed Bridge Deck

One of the promising applications of FRP bar-reinforced UHPC structures is their use as bridge decks, because FRP bars and UHPC can significantly reduce the self-weight of the bridge deck and improve its durability. As the density of FRP is only 1/7–1/5 times that of steel, the self-weight of the FRP bar-reinforced UHPC deck is lower than that of a steel bar-reinforced one, and the cost of labor would be reduced during the construction. For bridges such as long-span cable-stayed bridges and suspension bridges, the construction cost of which is very sensitive to the deadweight of the deck, FRP bars can help reduce the deadweight of the bridge deck and increase the bridge span. Moreover, considering the highly insulating properties of FRP bars, the FRP bar-reinforced UHPC bridge deck can be applied in railway bridges, where the inductive impedance caused by steel meshes results in a poor transmission performance through the track circuit [16].

**Figure 1:**
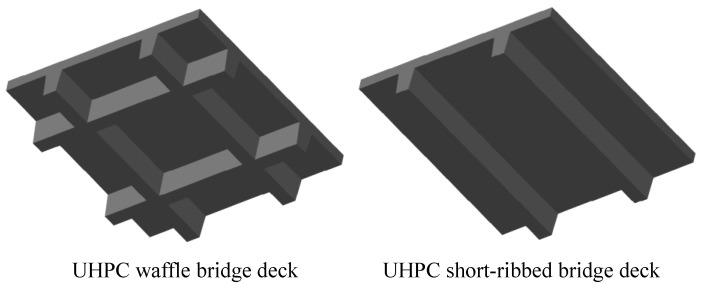
UHPC waffle bridge deck and short-ribbed bridge deck.

A super light FRP bar-reinforced UHPC waffle movable bridge deck was developed by Baghi et al. [17], and was used to replace the grid steel deck used in traditional movable bridges. In this study, inspired by the study by Qiu et al. [18], who successfully reduced the self-weight of the steel bar-reinforced waffle deck by eliminating the transverse rib, the FRP bar-reinforced UHPC short-ribbed bridge deck was proposed, as shown in Figure 1.

Depending on the types of fibers, different FRP bars have distinctly different mechanical characteristics [4]. Recently, the application of structure reinforcement using basalt FRP (BFRP) bars has developed quickly, because BFRP bars have excellent insulating properties and high economical advantage compared to other types of FRP bars [19]. Therefore, BFRP bars were adopted in the proposed FRP bar-reinforced UHPC short-ribbed bridge deck.

### 2.2. Behavior of the Proposed Bridge Deck under Concentrated Load

**Figure 2:**
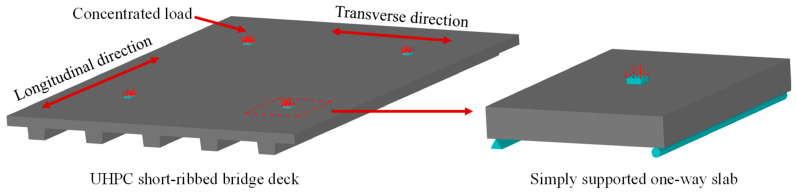
UHPC short-ribbed bridge deck and one-way slab.

The bridge deck is at risk of a punching shear failure when the concentrated load is applied by a wheel [20,21]. Therefore, the motivation of this paper is to evaluate the behavior of the proposed bridge deck under concentrated load, as shown in Figure 2. However, laboratory tests on the prototypes of the UHPC short-ribbed bridge deck are expensive and time-consuming. With the aim to simplify the specimen design and the test setup, finite element analysis with Abaqus software [22] was conducted first, to determine the deformation, stress and strain state of the deck under concentrated load.

**Figure 3:**
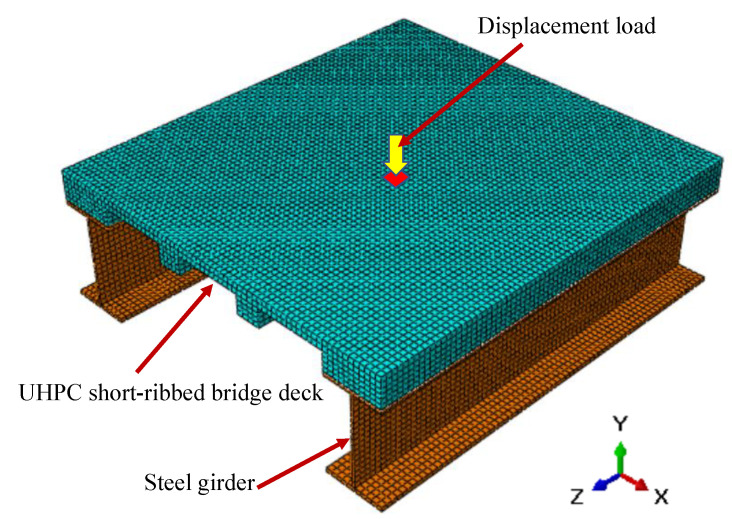
3D solid finite element model.

**Figure 4:**
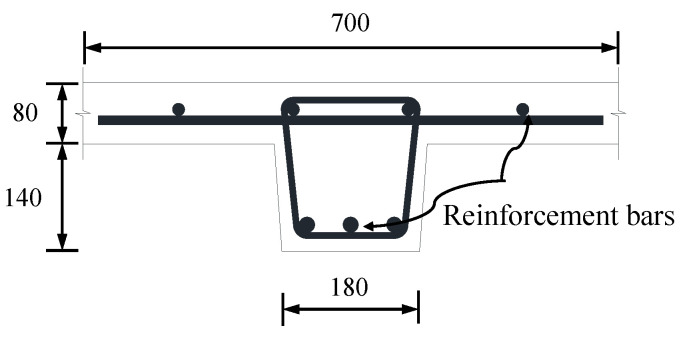
Cross section of the UHPC short-ribbed bridge deck (Unit: mm).

The 3D solid finite element model of a portion of the UHPC short-ribbed bridge deck is shown in Figure 3. The deck was of the same dimensions as that reported by Qiu et al. [18] (Figure 4), and it was supported by two steel I-girders. UHPC and steel materials were modeled as elastic materials, with the elastic moduli of 44.3 GPa and 210 GPa, respectively. The composite action between the deck and steel girders was modeled by tie constraint [21]. The bottom flanges of the steel girders were fixed. A displacement load of 1 mm was applied to a local area on the middle position of the deck surface.

**Figure 5:**
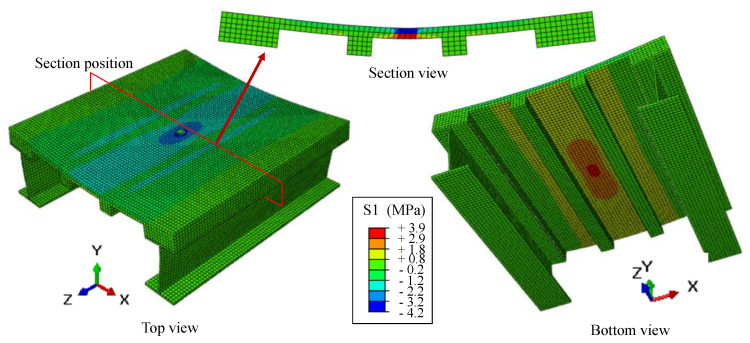
Results of the finite element model.

Figure 5 shows the contours of transverse stress in the x direction on the bridge deck, using a deformation scale factor of 100. The bridge deck was bent in the transverse direction. Significant stress was observed in the middle slab between the short ribs due to the concentrated load, while the stress in the ribs was close to zero. A section view of the slab showed this more clearly. Moreover, the bottom surface of the slab was in tension and the upper surface was in compression, indicating that the slab was generally positive bending. It was concluded by the numerical results that the slab between the short ribs worked as a one-way slab. The reason is that the slab was supported by the ribs in the longitudinal direction, while its length was much greater than the span between the ribs.

According to the simulation results, the slab between the short ribs was the most vulnerable part of the proposed deck under concentrated load, which suggested the need to investigate the behavior of this one-way slab under concentrated load. Many studies have found that the main factors that affect the punching shear capacities of slabs are concrete strength, slab thickness, loading area, flexural reinforcement, and boundary conditions [23,24]. The prediction methods for the punching shear capacities of steel bar-reinforced concrete slabs can be found in existing design codes, including ACI 318-19 [25], CAN/CSA A2 3.3-04 [26] and JSCE 2010 [27]. For FRP bar-reinforced concrete slabs, several design guides [5,6,7] provide prediction methods by modifying those employed for steel bar-reinforced concrete slabs after considering the difference sin material properties between steel bars and FRP bars. In addition, Matthys and Taerwe [28], Ospina et al. [29] and EI-Gamal et al. [20] also proposed modified prediction models by utilizing more experimental data. However, the existing design methods are intended for NC slabs, and few studies in the literature have focused on FRP bar-reinforced UHPC slabs [17]. In addition, the behavior of the FRP bar-reinforced UHPC slabs under concentrated load has not been studied. Therefore, the behavior of one-way UHPC slabs with BFRP bars under concentrated load was investigated. The slabs were simply supported, as shown in Figure 3. The effect of loading area, slab thickness and the type of reinforcements was studied. The failure process of slabs was analyzed, and the punching shear capacities were evaluated via the punching shear models presented in the literature.

## 3. Experimental Program

### 3.1. Materials

**Figure 6:**
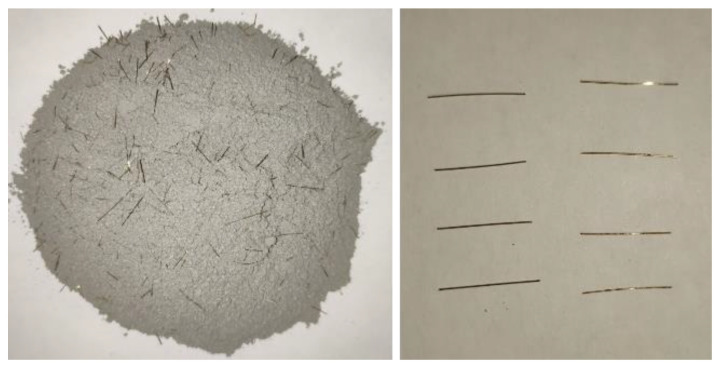
UHPC premix and straight steel fibers.

A commercial UHPC premix was adopted in this study, as shown in Figure 6. The water-to-binder ratio was 0.18, and the steel fibers content was 2% by volume. The length of steel fibers was 13 mm, the diameter was 0.2 mm, and the length-diameter ratio was 65. Other details of the mixture proportion were unclear.

For measuring the compressive strength of UHPC, three standard 100-mm concrete cubes were cast and tested according to the code DBJ 43/T325-2017 [30]. The compressive strength was determined to be 132.42 MPa. Moreover, the elastic modulus (*E_c_*) and the tensile strength (*f_t_*) of UHPC were calculated according to Equation (1) and Equation (2) in the code DBJ 43/T325-2017, respectively. The values were calculated to be 44.32 GPa and 6.22 MPa, respectively.

(1) $$E_{c}=10^{5}/(1.5+100/f_{cu})$$

(2) $$f_{t}=0.047f_{cu}$$

**Figure 7:**
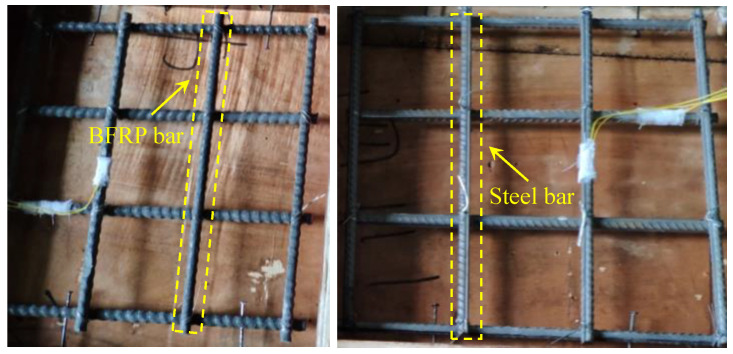
Photos of BFRP bars and steel bars.

BFRP bars and HRB335 steel bars were used as reinforcements in UHPC slabs, as shown in Figure 7. The diameter (*d_r_*) of all the bars was 10 mm. The material properties of the bars provided by the supplier were summarized in Table 1.

### 3.2. Specimens

**Figure 8:**
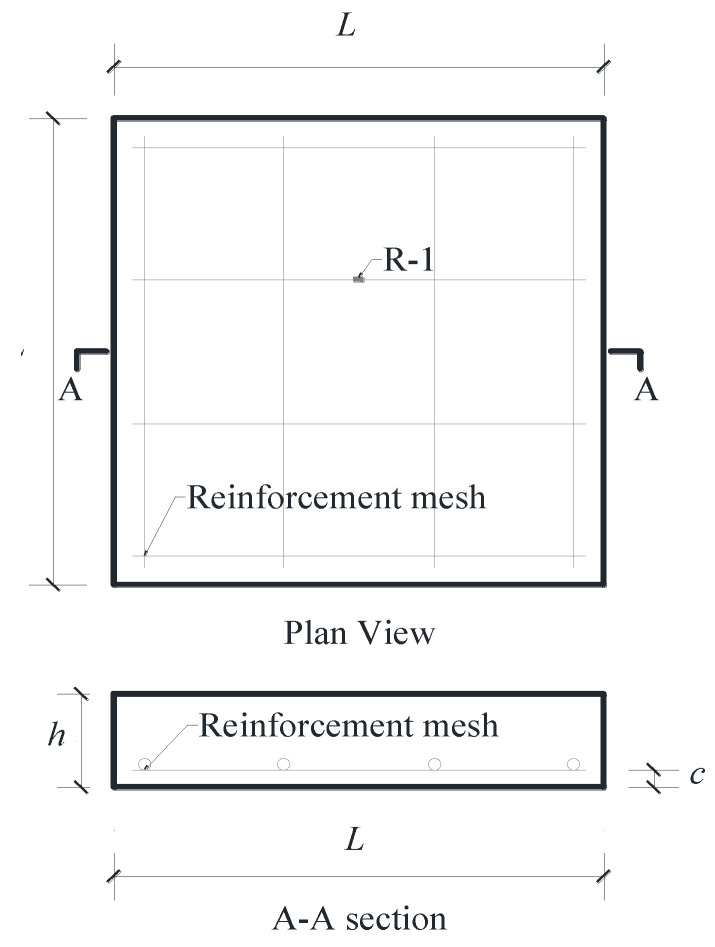
Geometric details of UHPC slabs.

As shown in Table 2, four UHPC slabs were fabricated and tested, among which one was reinforced with steel bars and three were reinforced with BFRP bars. The reinforcement arrangements are shown in Figure 8. The dimensions of the slabs were 400 mm × 400 mm × 80 mm or 400 mm × 400 mm × 100 mm. BFRP bars and steel bars were arranged in a mesh pattern. The cover thickness from the slab bottom to the bottom of the lower layer of the reinforcements was 15 mm. In all specimens, shear reinforcement was not provided, according to the design of UHPC slabs reported by Qiu et al. [18].

The test parameters were the loading area, slab thickness and the type of reinforcements. The first letter in the specimen label represented the type of reinforcements (with “S” for steel bars and “B” for BFRP bars), and the following two numbers are the slab depth and the side length of the loading area, respectively. The influence of the loading area was studied through tests on the B80-30 and B80-60 specimens. The B80-60 and B100-60 specimens were used to analyze the effect of increasing the slab thickness, while the influence of the reinforcement type was investigated via the B80-30 and S80-30 specimens.

The BRFP or steel reinforcement mesh was fixed in the wood mold. UHPC was cast from the top and center of the mold, and no mechanical vibration was used during the placement. Only a rubber hammer was used to tap the side of the mold to eliminate air bubbles. The UHPC slabs and cube specimens were cast in the same batch and cured under the same condition, such that no steam curing was applied.

### 3.3. Test Setup

**Figure 9:**
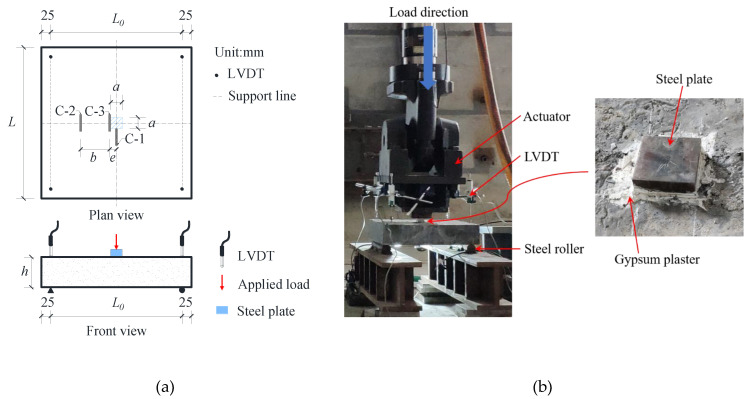
Test setup: (**a**) schematic diagram; (**b**) photo.

As shown in Figure 9, all the slabs were simply supported. A computer-controlled servo-hydraulic 200ton MTS actuator was used to apply the load to a rectangular steel plate on the slab. The loading speed was controlled to be lower than 10 kN/min. The load data were recorded automatically by the computer. A layer of high strength gypsum plaster was placed between the slab and the rectangular steel plate, so that the load was evenly distributed under the steel plate. In each specimen, a strain gauge was mounted on the middle of the reinforcement near the loading area, which was labeled R-1, as shown in Figure 8. The concrete strain gauges (C-1, C-2, C-3) parallel to the support were attached to the slab bottom, as shown in Figure 9. The deflection of the center of the slab was measured through four linear variable differential transformers (LVDTs), which were mounted on the actuator. Moreover, the cracking load and crack propagation process were recorded during the test. The bottom surface of the specimen was checked after each increase of 5 kN in the load.

## 4. Experimental Results

### 4.1. Failure Modes

**Figure 10:**
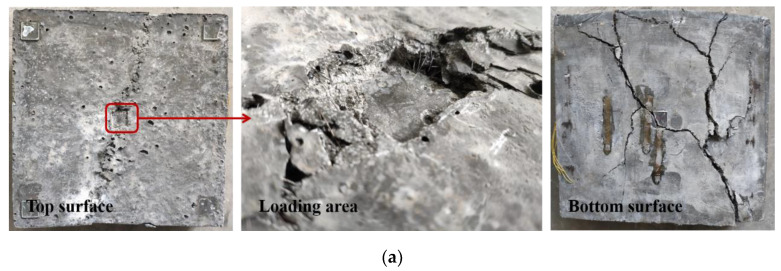

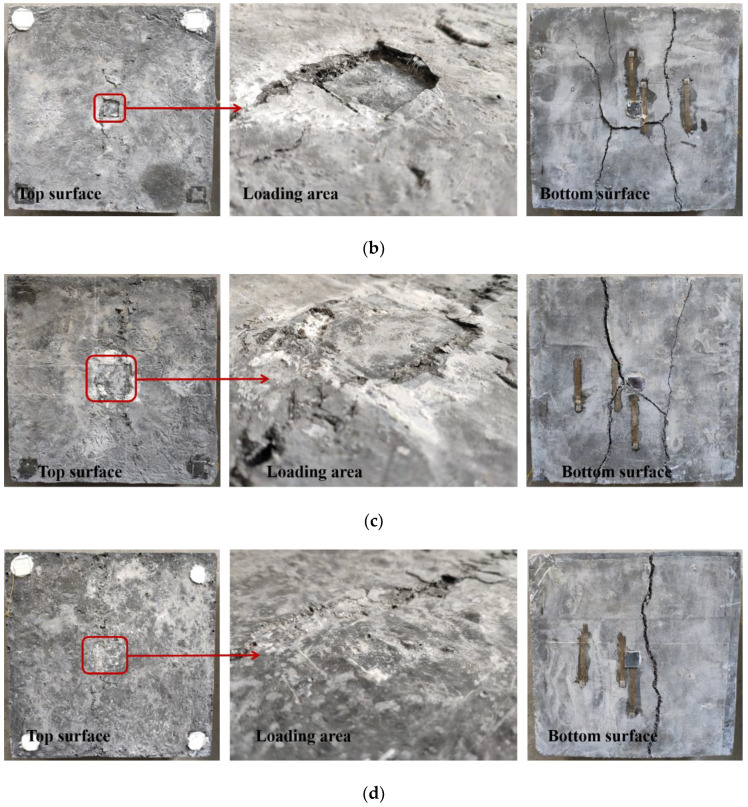
Failure modes: (**a**) S80-30; (**b**) B80-30; (**c**) B80-60; (**d**) B100-60.

**Figure 11:**
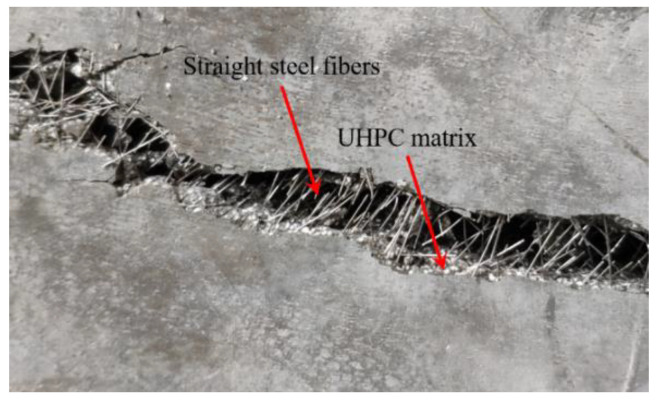
Steel fibers at the cracks.

With the load increasing, the deformation of the specimen gradually increased. The initiation and propagation of concrete cracks led to the failure of the specimen. The failure modes of the slabs, from the top and bottom views, are shown in Figure 10. In all one-way UHPC slabs, the supported sides of the slab were slightly tilted upward, and one or two main cracks parallel to the support were observed. Figure 11 shows that the steel fibers at the crack were pulled out from the UHPC matrix, indicating the fiber bridging effect at the crack, and the excellent post-cracking property of UHPC. Meanwhile, on the bottom surface of the UHPC slabs, most of the cover did not spall off, which often occurs in NC slabs under punching shear load [31,32]. There were fewer cracks on the top surface of the slab than on the bottom surface, and significant concrete crushing appeared on the top surface. In addition, the loading areas on the top surfaces of the S80-30 and B80-30 slabs were significantly depressed, and the depths of the depressions were measured to be 5.2 mm and 2.9 mm, respectively.

**Figure 12:**
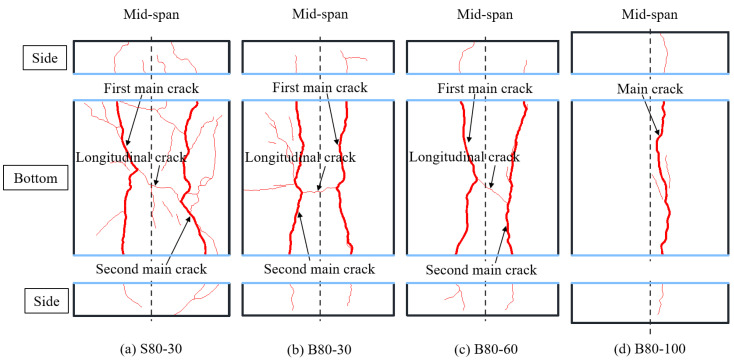
Crack patterns of UHPC slabs.

Cracks on the bottom surface and both sides of the slab were drawn, as shown in Figure 12. It was found that the first main cracks of all the slabs were initiated under the location of the concentrated load. The first main crack was defined as the crack that first appeared on the bottom surface of the slab during loading. Then, the crack propagated in the transverse direction parallel to the support, and extended up to the slab’s mid-height. Two main cracks were formed on the bottom surfaces of the B80-30, B80-60 and S80-30 slabs. Thereafter, the main cracks were connected by a longitudinal crack just under the loading area. It can be observed from the side view of the UHPC slab that both the main cracks were flexural-shear cracks. The cracks were approximately vertical on the bottom, and the inclination angle of the cracks gradually decreased as they extended upward. However, only one main crack occurred in the B100-60 slab, and no punching shear surface was formed. This crack was approximately parallel to the support, and was located under the edge of the rectangular steel loading plate. It can be seen that the crack patterns are not characteristic of punching shear failure. The main reason for this is that the slabs in this study were one-way slabs. Moreover, based on the tests on two-way slabs under concentrated load, Matthys and Taerwe [28] also pointed out a strong interaction between flexural and shear effects. Punching shear failure is close to flexural failure, especially for FRP bar-reinforced NC slabs with low reinforcement ratios [28]. In addition, it was determined by comparing the failure modes of specimens B80-60 and B100-60 that the flexural effect was more obvious as the slab thickness increased. Therefore, the crack pattern of the one-way UHPC slab under concentrated load was significantly affected by the flexural behavior of its cross section.

According to the crack patterns of the B80-30 and B80-60 slabs, the number of cracks decreased as the loading area increased. As for the effect of the type of reinforcement, the comparison between the crack patterns of the S80-30 slab and the B80-30 slab showed that a denser crack distribution was observed in the steel bar-reinforced UHPC slab. Yoo et al. [10,11] reported a similar observation for the difference in crack spacing between GFRP bar-reinforced and steel bar-reinforced UHPC beams. The reason was assumed to be that GFRP bars have less longitudinal rigidity than steel bars.

### 4.2. Load-Deflection Relationship

**Figure 13:**
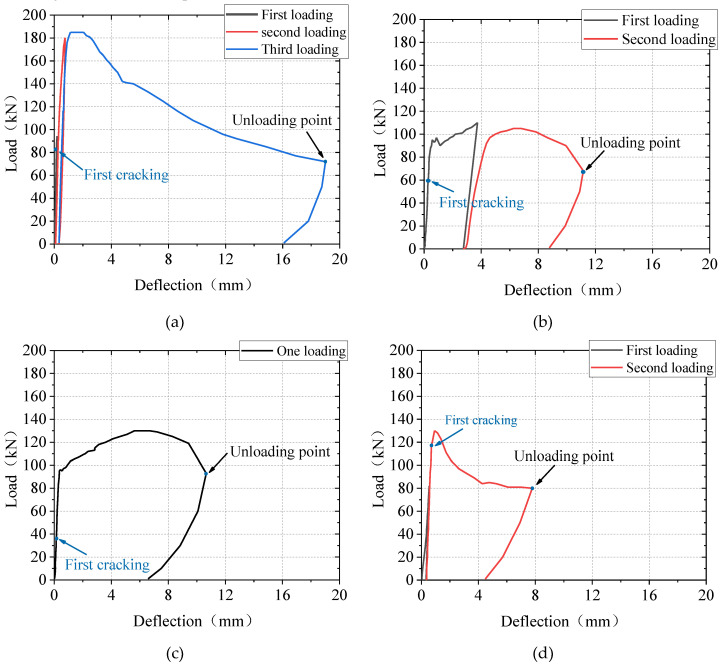
Load-deflection curves: (**a**) S80-30; (**b**) B80-30; (**c**) B80-60; (**d**) B100-60.

Figure 13 shows the measured load and deflection curves. The loading plate slid out easily during the test, which led to the test’s suspension. Therefore, some slabs were repeatedly loaded. The initial stiffnesses of the load-deflection curves for all the slabs were high. At the same time, after the formation of the first main crack, the stiffness of all the test slabs did not significantly change immediately. This was different from the results derived from the punching shear tests on FRP bar-reinforced NC slabs, whose stiffness under concentrated load significantly decreased after the development of the first crack, due to the insufficient tensile strength and ductility of NC [31,32]. Therefore, it can be concluded that the relatively higher stiffness of the one-way UHPC slabs after initial cracking was a result of the strain-hardening response of UHPC, which was caused by the fiber bridging effect at the crack surfaces. With the propagation of cracks, only the capacity of the B100-60 slab dropped suddenly, while the load-deflection curves of the B80-30 and B80-60 slabs exhibited plateaus after the peak load. A short plateau was also observed in the curve of the S80-30 slab. Specimens were unloaded at about 70 kN, and they all exhibited good deformation recovery capacities. The unloading stiffness of the B80-30 slab was similar to that of the S80-30 slab, and the recovered displacement was about 3 mm in both slabs. Wang et al. [32] has pointed out that the BFPR bar-reinforced NC slab under concentrated load exhibited a better deformation recovery capacity than the steel bar-reinforced NC slab, but this was inconsistent with the result in this study. The geometry of the specimens may affect the results. The specimens in this study are smaller than those in the study of Wang et al. [32]. As a result, in addition to the effect of material ductility, the bond between the reinforcement bars and the UHPC will have a non-negligible effect on the ductility of the specimens. Due to the wider cracks in the BFRP bar-reinforced UHPC slabs, the damage to the bond would be greater in those slabs than in the steel bar-reinforced UHPC slab, which leads to the reduced deformation recovery ability. This may also be the reason for the poor ductility of the B100-60 specimen.

**Figure 14:**
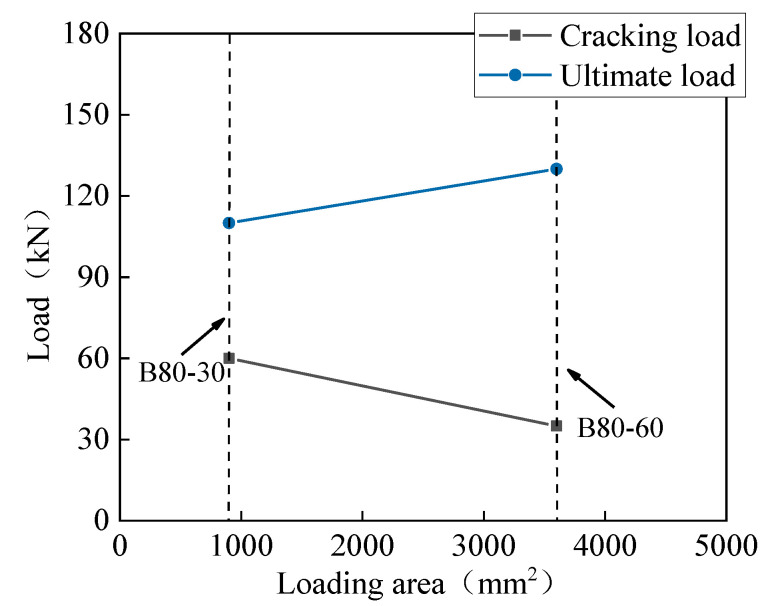
Effect of loading area on the cracking and peak load.

**Figure 15:**
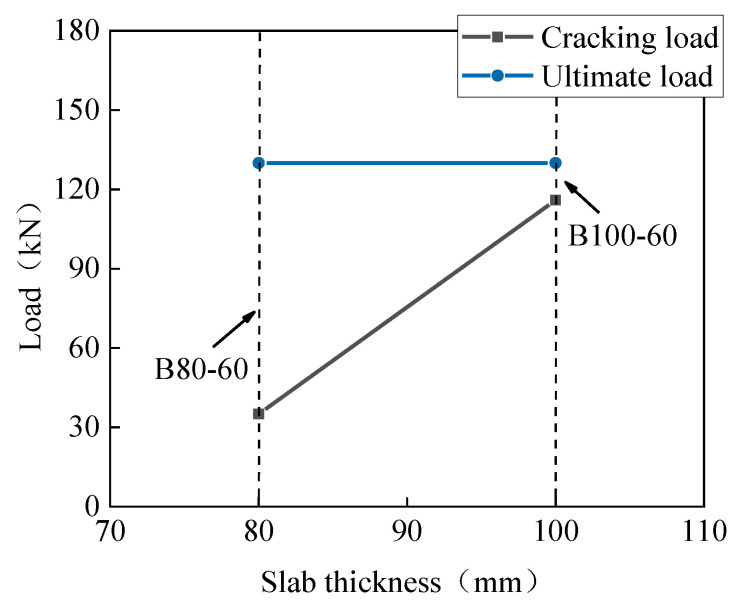
Effect of slab thickness on the cracking and peak load.

Table 3 summarizes the cracking and peak load of the slabs, as well as the corresponding deflections. Under the conditions of the same loading area and reinforcement ratio, the cracking load and the peak load of the B80-30 slab were 0.73 times and 0.59 times that of the S80-30 slab, respectively. The effect of the loading area on the cracking load and peak load is shown in Figure 14. The peak load of the B80-60 slab was 18.2% higher than that of the B80-30 slab, while its cracking load was 42% smaller than that of the B80-30 slab. The peak load of the one-way BFRP bar-reinforced UHPC slabs increased with the increase in the loading area. Figure 15 represents the influence of slab thickness on the cracking and peak load of one-way UHPC slabs. The peak loads of the slabs B100-60 and B80-60 were similar, while the cracking load increased with the slab thickness. The reason for this could be that, though the cracking moment of the B100-60 slab was larger due to the greater slab thickness, its reinforcement ratio was smaller, and the development of the first main crack was sudden.

### 4.3. Strain Results

**Figure 16:**
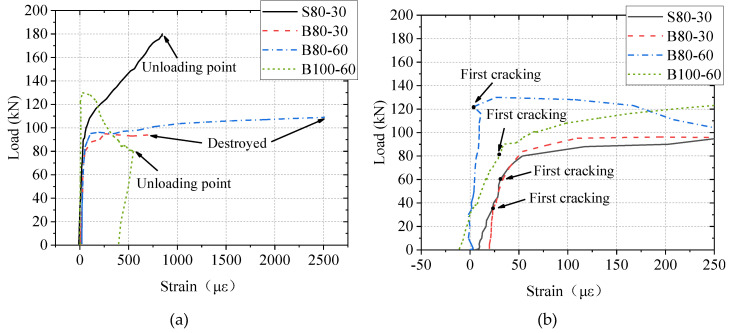
Results of strain in bars: (**a**) overall strain; (**b**) initial strain.

As shown in Figure 16, in all the slabs, the ultimate strain of the BFRP bar or the yielding strain of the steel bar was not reached, meaning that the BFRP bars and the steel bars were not fractured. The measured strain of the reinforcements (steel bars/BFRP bars) in the UHPC slabs increased linearly and slowly with the load in the early loading stage, when most of the stress in the tension zone was carried by the UHPC. This did not change even after the occurrence of the first main crack, as shown in Figure 16b. Researchers [31,32] reported that after the formation of cracks, the reinforcement strain increased significantly in FRP bar-reinforced normal concrete slabs under concentrated load. This difference between slabs using normal concrete and UHPC was attributed to the strain-hardening response of UHPC. Then, at the load of about 95 kN, the strain of the reinforcements in the B80-30 and B80-60 slabs increased significantly. However, the growth rate of strain in the S80-30 slab was almost constant after the first main crack was formed at the load of about 82 kN. The difference in the strain growths of the two types of bars was because the BFRP bars have a smaller elastic modulus than steel bars, and a larger strain was required in the BFRP bars to manifest the same value of stress reduction resulting from the cracking of UHPC.

**Figure 17:**
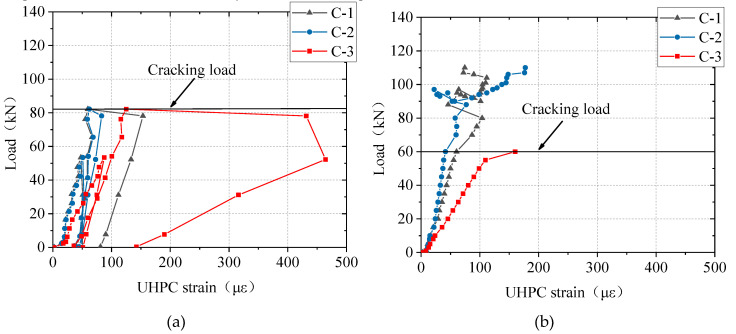

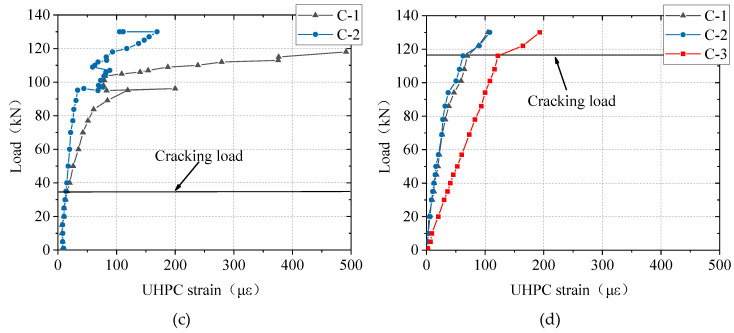
Results of strain tests in UHPC: (**a**) S80-30; (**b**) B80-30; (**c**) B80-60; (**d**) B100-60.

Figure 17 illustrates the results of strain in UHPC. There was a clear change in the growth rate of the UHPC’s strain at the cracking load. The measured strain from the C-3 strain gauge was the largest. Its value was about 150 με at the cracking load.

### 4.4. Ductility

In addition to the loading capacity, the ductility of the slab should also be assessed in the design. For traditional steel bar-reinforced NC members, the ductility index is the ratio of the deflection at the peak load to that at the yielding load. As BFRP bars exhibit an almost linear elastic behavior in tension until reaching failure, the traditional ductility index is not applicable to FRP bar-reinforced members. Therefore, a ductility index (*μ_e_*) was suggested by Naaman and Jeong [33], as shown in Equation (3). Here, this index was used to evaluate the ductility of all the UHPC slabs in this study.

(3) $$\mu_{e}=\frac{1}{2}\left(\frac{E_{tot}}{E_{el}}+1\right)$$

where *E_tot_* is the total energy, and *E_el_* is the elastic energy.

**Figure 18:**
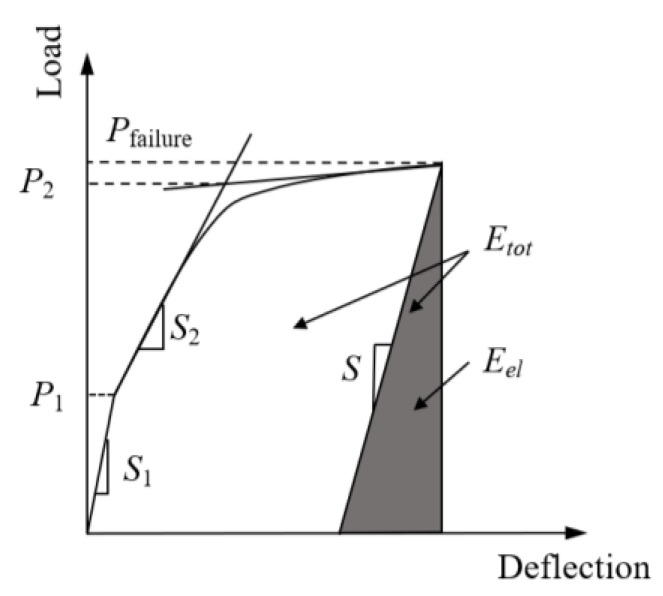
Definition of ductility index [33].

For calculating the elastic energy and total energy, the unloading curve at the failure load was required (Figure 18). The slope of the unloading curve (*S*) was assumed to be the weighted average slope of the two initial straight lines of the load-deflection curve [33]. Then, the slope of the unloading curve is calculated from Equation (4).

(4) $$S=\left[P_{1}S_{1}+(P_{2}-P_{1})S_{2}\right]/P_{2}$$

where *P*_1_ is the cracking load and *P*_2_ is the load, as shown in Figure 18; *S*_1_ and *S*_2_ are the corresponding slopes. Then, the total energy was calculated by integrating the corresponding area in Figure 18 with Equation (5), and the elastic energy was calculated as (*P*_failure_^2^/*S*/2).

(5) $$E_{tot}=\int_{0}^{\Delta_{u}}P\left(\Delta \right)\times \Delta d\Delta$$

**Figure 19:**
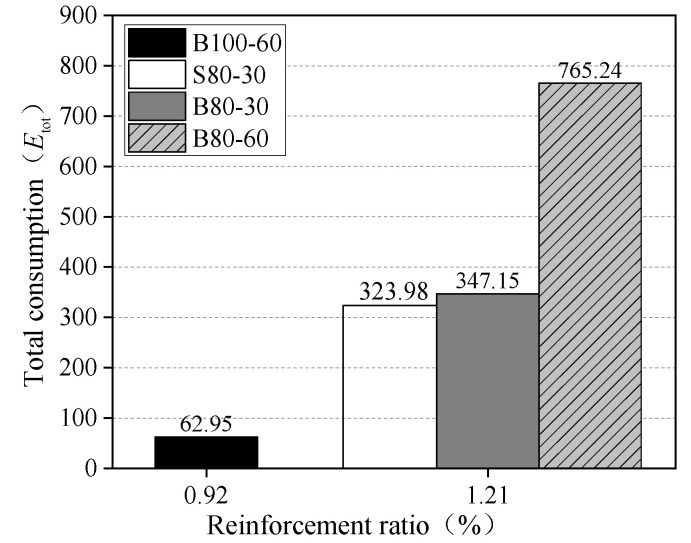
Total energy.

**Figure 20:**
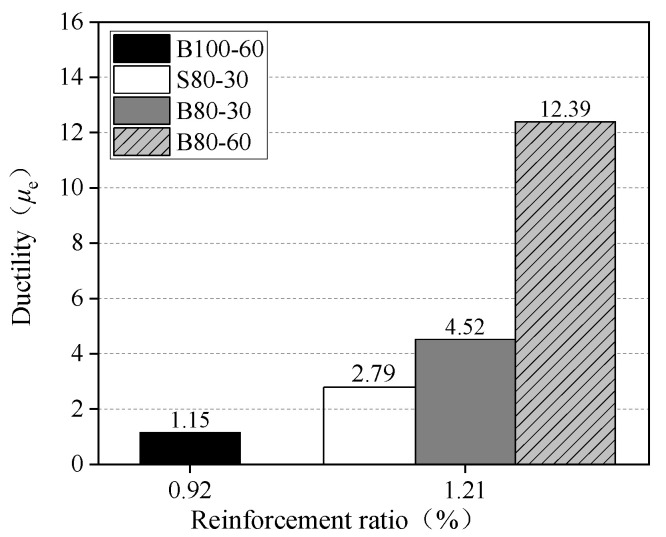
Ductility index.

The calculation results are summarized in Table 3. The results of the total energy and the ductility index are presented in Figure 19 and Figure 20, respectively. The total energy of the B80-60 slab was about 2.20 times that of the B80-30 slab, and the ductility index was about 2.74 times greater. Hence, it was determined that the slab has a greater ductility under a larger loading area. The total energy of the B80-30 slab was 7.2% higher than that of the S80-30 slab, while the ductility index of the B80-30 slab was 62% higher. Using BFRP bars as reinforcement in the UHPC slabs was conducive to the improvement of ductility. The total energy and ductility index of the B100-60 slab were the lowest because the failure mode was different, due to its lower reinforcement ratio. Therefore, increasing the reinforcement ratio of the BFRP bars was beneficial to the total energy and ductility of one-way BFRP bar-reinforced UHPC slabs.

## 5. Prediction of Punching Shear Capacities

At present, there are no prediction methods for the punching shear capacity of a one-way slab in the design codes or guides, so the methods for two-way slabs were considered instead. The punching shear capacity of the S80-30 slab was calculated using the prediction methods for two-way steel bar-reinforced NC slabs taken from certain design codes, including ACI 318-19 [25], CAN/CSA A2 3.3-04 [26] and JSCE 2010 [27]. For FRP bar-reinforced NC slabs, the existing prediction methods for punching shear capacity are generally obtained by modifying those used for steel bar-reinforced NC slabs after considering the differences in the material properties of steel bars and FRP bars. As shown in Table 4, prediction methods for two-way FRP bar-reinforced NC slabs in the current design guides, including ACI 440.1R-15 [5], CAN/CSA S806-12 [6] and JSCE 1997 [7], were used in this study to calculate the punching shear capacities of the one-way BFRP bar-reinforced UHPC slabs. In addition, the theoretical models for FRP bar-reinforced NC slabs proposed by Matthys and Taerwe [28], Ospina et al. [29] and EI-Gamal et al. [20] were also adopted.

The ratios of the predicted punching shear capacities to the experimental ones (*P*_exp._) are summarized in Table 5. Note that when calculating the punching shear capacity of the S80-30 slab by Equations (12)–(14), *E_f_* was replaced with *E_s_*. In Equations (6)–(14), the cylinder compressive strength of UHPC was required. As experimentally indicated by Graybeal and Davis [34], the ratio of the cylinder compressive strength of UHPC to the cube compressive strength can be taken as 1.0. Therefore, the cube compressive strength of UHPC was used in the calculation.

The results show that ACI 318-19 [25], CAN/CSA A23.3-04 [26] and JSCE 2007 [27] significantly underestimated the punching shear capacity of the S80-30 slab. The punching shear capacities of all the one-way BFRP bar-reinforced UHCP slabs were significantly underestimated by ACI 440.IR-15 [5] and JSCE 1997 [7]. The predicted capacities of CAN/CSA S806-12 [6] were slightly higher than those of ACI 440.IR-15 and JSCE 1997, but the punching shear capacities of the test slabs were still underestimated. To ensure the reasonable safety of the prediction of the punching shear capacity, the predictions of the design codes and guides must be conservative. Thus, it is reasonable that the punching shear capacity of the slab was underestimated by the design codes and guides. On the other hand, the existing design methods are intended for NC slabs. The strength of the UHPC slab would be underestimated, because the fact that the strength of UHPC is greater than that of NC cannot be considered in these methods. As a result, the safety provided by these methods for the two-way NC slabs exceeds the commonly accepted bounds.

The models of Matthys and Taerwe [28] and Ospina et al. [29] were also found to give conservative predictions, because both methods were based on the research results of two-way FRP bar-reinforced NC slabs. Therefore, it can be concluded that the prediction method for two-way FRP bar-reinforced NC slabs is not applicable to one-way FRP bar-reinforced UHPC slabs.

The predicted capacities of the model from EI-Gamal et al. [20] were higher than those of Equations (12) and (13). The boundary conditions of the slab have a significant influence on the punching shear capacity of the slab [23,35]. Thus, the punching shear capacities of the one-way slab and two-way slab are different. Only EI-Gamal et al. [20] carried out tests on one-way FRP bar-reinforced bridge deck slabs under concentrated load, and the proposed model provided better predictions of the punching shear capacity. Therefore, it was determined in this study that this model is more suitable for calculating the punching shear capacities of one-way BFRP bar-reinforced UHPC slabs. However, UHPC has an excellent strain-hardening characteristic, which makes their prediction model more conservative. In addition, the model proposed by EI-Gamal et al. [20] overestimated the punching shear capacity of the B100-60 specimen. This could be attributed to the change in the failure mode of the B80-60 specimen, which is out of the application range of the model.

## 6. Conclusions

In this study, an innovative BFRP bar-reinforced UHPC short-ribbed bridge deck was proposed, and investigated via tests on one-way BFRP bar-reinforced UHPC slabs under concentrated load. The following conclusions were drawn.

- Compared with the steel bar-reinforced UHPC slab, the cracks in the BFRP bar-reinforced UHPC slab were wider, with larger spacings after the failure of the slab. Moreover, the cracking load and the peak load of the BFRP bar-reinforced slab were 0.73 times and 0.59 times those of the steel bar-reinforced one, respectively. Though the use of BFRP bars led to a reduction of the capacity of the one-way UHPC slab under concentrated load, it is favorable for improving the ductility of the slab.
- When the BFRP reinforcement ratio increased from 0.92% to 1.21%, or the loading area became four times larger, the ductility index of the slab increased about two times, while the ultimate capacity of the slab was insignificantly affected. It was noted that a sufficient reinforcement ratio was a prerequisite for achieving high ductility in BFRP bar-reinforced UHPC slabs.
- The design methods for two-way FRP bar-reinforced NC slabs in ACI 440.IR-15 [5], CAN/CSA S806-12 [6] and JSCE 1997 [7] significantly underestimated the capacities of the tested one-way UHPC slabs. The model proposed by EI-Gamal et al. [20], based on test results of one-way FRP bar-reinforced NC slabs, was found to be suitable for evaluating the punching shear capacities of one-way BFRP bar-reinforced UHPC slabs. However, the model failed to consider the unique strain-hardening characteristics of UHPC, which led to conservative predictions. More experiments on this type of slab are suggested.

The results can be a reference for the design and application of BFRP bar-reinforced UHPC short-ribbed bridge deck slabs with the desired punching shear capacity. However, the experimental results of this paper represent a relatively small database, and thus, the above conclusions are qualitative. Additional experimental studies will be conducted in the future.

## Figures and Tables

**Table 1:** Properties of BFRP bars and steel bars.

Reinforcement Type	*d_r_* (mm)	*f_u_* (MPa)	*f_y_* (MPa)	*E* (GPa)	*ε_fu,s_* (mm/mm)	*ε_fu,BFRP_* (mm/mm)
BFRP bar	10	810	-	50	0.0017	-
Steel bar	10	-	345	200	-	0.0162

Note: *f_u_* and *ε_fu,BFRP_* are the ultimate strength and ultimate strain of BFRP bars, respectively; *f_y_* and *ε_fu,s_* are the yield strength and yield strain of steel bars, respectively; *E* is the elastic modulus.

**Table 2:** Details of UHPC slabs.

Specimens	*L*	*L_0_*	*h*	*a*	*d*	*b*	*e*	*ρ_f_*	Reinforcement Type	Reinforcement Configuration
	(mm)							(%)		
S80-30	400	350	80	30	55	70	30	1.21	Steel	10 mm @ 110 mm
B80-30	400	350	80	30	55	70	30	1.21	BFRP	10 mm @ 110 mm
B80-60	400	350	80	60	55	70	30	1.21	BFRP	10 mm @ 110 mm
B100-60	400	350	100	60	75	70	30	0.92	BFRP	10 mm @ 110 mm

Note: *L* is the side length of the slab; *L_0_* is the effective span of the slab; *h* is the slab depth; *a* is the side length of loading area; *d* is the effective depth of the slab (taken as the average value of the effective depth of the cross section in the longitudinal and transversal directions); *ρ_f_* is the reinforcement ratio; *b* and *e* are the positions of strain gauges.

**Table 3:** Experimental and calculation results.

Specimens	First Cracking		Ultimate State		Ductility Calculation		
	*P_cr_* (kN)	Δ*_cr_* (mm)	*P_u_* (kN)	Δ*_u_* (mm)	*E_tot_* (N.m)	*E_el_* (N.m)	*μ_e_*
S80-30	82	0.18	185	2.05	323.97	70.64	2.79
B80-30	60	0.29	110	3.74	347.15	43.16	4.52
B80-60	35	0.14	130	6.67	765.24	32.16	12.39
B100-60	116	0.70	130	0.92	62.95	48.12	1.15

Note: *P_cr_* is the cracking load and Δ*_cr_* is the corresponding deflection; *P_u_* is the peak load and Δ*_u_* is the corresponding deflection; *E_tot_* is total energy; *E_el_* is elastic stored energy; *μ_e_* is the ductility index.

**Table 4:** Punching shear equations of concrete slabs.

References	Equation	Type of Reinforcements	Type of Slabs	Equation No.
ACI 318-19 [25]	$\begin{array}{l} V_{c}=0.33\sqrt{f_{c}^{’}}b_{0}d\\ b_{0}=4(a+d),\sqrt{f_{c}^{’}}\leq 8.3\mathrm{MPa}\; \end{array}$	Steel bars	Two-way	(6)
CAN/CSA A23.3-04 [26]	$\begin{array}{l} V_{c}=V_{\min}=\left\{\begin{array}{l} \left(1+\frac{2}{\beta_{c}}\right)0.19\lambda \varphi_{c}\sqrt{f_{c}^{’}}b_{0}d\\ \left(\frac{\alpha_{s}d}{b_{0}}+0.19\right)\lambda \varphi_{c}\sqrt{f_{c}^{’}}b_{0}d\\ 0.38\lambda \varphi_{c}\sqrt{f_{c}^{’}}b_{0}d \end{array}\right\}\\ b_{0}=4(a+d),\sqrt{f_{c}^{’}}\leq 8\mathrm{MPa} \end{array}$	Steel bars	Two-way	(7)
JSCE 2010 [27]	$\begin{array}{l} V_{c}=\beta_{d}\beta_{p}\beta_{r}f_{pcd}b_{0}d\\ \beta_{d}={\left(1000/d\right)}^{1/4}\leq 1.5,\beta_{p}={\left(100\rho_{s}\right)}^{1/3}\leq 1.5,\\ \beta_{r}=1+1/(1+0.25u/d),\\ f_{pcd}=0.2\sqrt{f_{c}^{’}}\leq 1.2\mathrm{MPa},b_{0}=4a+2\pi (0.5d) \end{array}$	Steel bars	Two-way	(8)
ACI 440.1R-15 [5]	$\begin{array}{l} V_{c}=\frac{4}{5}\sqrt{f_{c}^{’}}b_{0}kd\\ k=\sqrt{2\rho_{f}n_{f}+{\left(\rho_{f}n_{f}\right)}^{2}}-\rho_{f}n_{f},n_{f}=E_{f}/E_{c},b_{0}=4(a+d) \end{array}$	FRP bars	Two-way	(9)
CAN/CSA S806-12 [6]	$\begin{array}{l} V_{c}=V_{\min}=\left\{\begin{array}{l} 0.028\lambda \left(1+\frac{2}{\beta_{c}}\right){\left(E_{f}\rho_{f}f_{c}^{’}\right)}^{1/3}b_{0}d\\ 0.147\lambda \left(\frac{\alpha_{s}d}{b_{0}}+0.19\right){\left(E_{f}\rho_{f}f_{c}^{’}\right)}^{1/3}b_{0}d\\ 0.056\lambda {\left(E_{f}\rho_{f}f_{c}^{’}\right)}^{1/3}b_{0}d \end{array}\right\}\\ b_{0}=4(a+d) \end{array}$	FRP bars	Two-way	(10)
JSCE 1997 [7]	$\begin{array}{l} V_{c}=\beta_{d}\beta_{p}\beta_{r}f_{pcd}b_{0}d\\ \beta_{d}={\left(1000/d\right)}^{1/4}\leq 1.5,\beta_{p}={\left(100\rho_{f}E_{f}E_{s}\right)}^{1/3}\leq 1.5,\\ \beta_{r}=1+1/(1+0.25u/d),\\ f_{pcd}=0.2\sqrt{f_{c}^{’}}\leq 1.2\mathrm{MPa},b_{0}=4a+2\pi (0.5d) \end{array}$	FRP bars	Two-way	(11)
Matthys and Taerwe [28]	$\begin{array}{l} V_{c}=1.36\frac{{\left(100\rho_{f}E_{f}/E_{s}f_{c}^{’}\right)}^{1/3}}{d^{1/4}}b_{0}d\\ b_{0}=4a+12d \end{array}$	FRP bars	Two-way	(12)
Ospina et al. [29]	$\begin{array}{l} V_{c}=2.77{\left(\rho_{f}f_{c}^{’}\right)}^{1/3}\sqrt{\frac{E_{f}}{E_{s}}}b_{0}d\\ b_{0}=4a+12d \end{array}$	FRP bars	Two-way	(13)
EI-Gamal et al. [20]	$\begin{array}{l} V_{c}=0.33\sqrt{f_{c}^{’}}b_{0}d\alpha \\ \alpha =0.62{\left(\rho E_{f}\right)}^{1/3}\left(1+8d/b_{0}\right),b_{0}=4(a+d) \end{array}$	FRP bars	One-way	(14)

Note: *V_c_* is the punching shear capacity (N); *f_c_^’^* is the compressive strength of the concrete (MPa); *b_0_* is the critical perimeter; a is the side length of the loading area; *d* is the effective depth of the slab; *ρ_f_* is the FRP reinforcement ratio; *ρ_s_* is the steel reinforcement ratio; *E_f_* is the modulus of elasticity of the FRP bars (MPa); *E_s_* is the modulus of elasticity of the steel bars (MPa); *n_f_* is the ratio of the modulus of elasticity of the FRP bars to the modulus of elasticity of concrete; *λ* is the factor to account for concrete density (*λ* = 1 in this study); *u* is the perimeter of the loading area (mm).

**Table 5:** Comparison between predicted and experimental punching shear capacities.

Specimens	*P*_exp._ (kN)	Comparison with Codes			Comparison with Theoretical Models		
		*P_ACI_*/*P*_exp._	*P_CSA_*/*P*_exp._	*P_JSCE_*/*P*_exp._	*P_Ospina_*/*P*_exp._	*P_MT_*/*P*_exp._	*P_EI-Gamal_*/*P*_exp._
S80-30	185.0	0.28 ^a^	0.23 ^a^	0.27 ^a^	0.75 ^b^	0.63 ^b^	0.70 ^b^
B80-30	110.0	0.24	0.41	0.29	0.63	0.67	0.78
B80-60	130.0	0.27	0.47	0.31	0.52	0.65	0.76
B100-60	130.0	0.38	0.69	0.47	0.97	0.95	1.20

Note: *P*_exp._ is the experimental punching shear capacity; symbol “^a^“ means that the design codes including ACI 318-19 [25], CAN/CSA A2 3.3-04 [26] and JSCE 2010 [27] were used for the S80-30 slab; symbol “^b^“ means that *E_f_* was replaced with *E_s_* in Equations (12)–(14).
